# A Systematic Literature Review and Bibliometric Analysis of Ophthalmology and COVID-19 Research

**DOI:** 10.1155/2022/8195228

**Published:** 2022-05-24

**Authors:** Ali Forouhari, Vahid Mansouri, Sare Safi, Hamid Ahmadieh, Amir Ghaffari Jolfayi

**Affiliations:** ^1^Ophthalmic Research Center, Research Institute for Ophthalmology and Vision Science, Shahid Beheshti University of Medical Sciences, Tehran, Iran; ^2^Gene Therapy Research Center, Digestive Diseases Research Institute, Shariati Hospital, Tehran University of Medical Sciences, Tehran, Iran; ^3^Ophthalmic Epidemiology Research Center, Research Institute for Ophthalmology and Vision Science, Shahid Beheshti University of Medical Sciences, Tehran, Iran

## Abstract

This review is proposed to summarize the updates on COVID-19 and ophthalmology along with the bibliometric features of articles that have been published since the beginning of the COVID-19 outbreak. The databases, including PubMed, Scopus, and Web of Science, were searched using “Coronavirus,” “COVID-19,” “SARS-CoV-2,” “pandemic,” “ophthalmology,” “ophthalmic,” and “eye” keywords. All published articles except commentaries, errata, and corrigenda up to April 2021 were included. Titles and abstracts were screened, and ophthalmology-focused articles were collected. The bibliographic information of the articles, such as the name and country of the first author, type of study, date of publication, language, and journal name, were extracted. Included studies were assessed using the Joanna Briggs Institute (JBI) critical appraisal checklist. After systematic searching, 2,669 distinct articles were screened by title/abstract, and 1,174 ophthalmology-focused articles were selected to be reviewed. Ophthalmology-focused publications accounted for less than 0.5 percent of the total COVID-19-related articles. Most of the articles were published in the Indian Journal of Ophthalmology, and the main publication type was “original article.” Almost 88% of the publications were in English. There was a decline in the publication rate during the initial months of 2021 compared with the middle and last months of 2020. Most of the publications were affiliated with the United States of America. However, Singapore and the United Kingdom were the countries with the highest number of publications after population adjustment. Furthermore, a comprehensive review on major topics including SARS-CoV-2 ocular tropism, ophthalmic manifestations, ocular complications due to COVID-19 treatment strategies, the pandemic effect on ophthalmology care and operations, myopia progression during the pandemic, and telemedicine was conducted.

## 1. Introduction

In December 2019, some suspected cases of a pneumonia-like disease were first identified in Wuhan, China. On 7^th^ January 2020, the World Health Organization (WHO) declared the appearance of a virus causing a severe acute respiratory syndrome, later called severe acute respiratory syndrome coronavirus 2 (SARS-CoV-2) [1]. On 13^th^ January, the first confirmed case of coronavirus disease 2019 (COVID-19) outside of China was noticed in Thailand, and then it progressively spread all over the world. One of the first clinicians who described the symptoms and signs of this pneumonia-like disease and played a role in starting COVID-19 global awareness was Dr. Wenliang Li, an ophthalmologist practicing in Wuhan, China [2]. Finally, on 11^th^ March, the WHO declared COVID-19 as a global pandemic, and almost 130 million approved cases and about 3 million deaths due to COVID-19 have been identified until April 2, 2021 [3].

The most prominent viral outbreaks during the recent century have shown different ocular manifestations [4]. Ophthalmic features of COVID-19 and probable ocular side effects of the drugs cause ophthalmologists to come into close contact with patients, making them prone to a high risk of virus contamination. Detection of virus particles in tears and ocular secretions has raised controversies about the transmission potency of droplets and aerosols during ocular examinations and surgeries. In addition, the high risk of virus spread during hospitalization and interventions has limited eye care, reduced ophthalmologic procedures, and postponed elective surgeries. In the lockdown state, telemedicine has gained more attention than before and has shown its efficacy to serve as a potential route for delivering medical information. Similarly, ophthalmology education in undergraduate and residency programs has changed to correspond better with this pandemic situation.

Considering the emergence of rapidly progressive evidence generation about SARS-CoV-2, this systematic summarize the bibliometric aspects of ophthalmology-focused COVID-19 scientific literature and discuss the findings in major issues of this field.

## 2. Methods

### 2.1. Literature Research

A comprehensive search syntax using MeSH and free text terms for PubMed and adapted text terms as appropriate for the other searched databases (Scopus and Web of Science) was developed through title/abstract/keywords (Supplementary Material 1). We searched all three databases from COVID-19 identification up to April 2021. A literature search was conducted investigating ophthalmologic aspects during the COVID-19 outbreak using combinations of the following keywords: “Coronavirus,” “COVID-19,” “SARS-CoV-2,” “pandemic,” “ophthalmology,” “ophthalmic,” and “eye” based on the Boolean logic model (using “AND” or “OR”).

### 2.2. Eligibility Criteria

We included all types of articles except commentaries, errata, and corrigenda. Among the identified ophthalmology-focused COVID-19 articles, we then manually classified them into 14 categories: original article, review article, letter to the editor, editorial, case report, note, practice guideline, report, correspondence, communications, short survey, abstract, conference paper, and book chapter.

### 2.3. Study Selection

The search results were uploaded to a reference manager software package (Endnote 20; Clarivate, Philadelphia, PA). Titles and abstracts were screened for relevance independently by 2 reviewers (AF and VM), with any disagreements being resolved by discussion and involvement of a third reviewer when necessary. All duplicate papers were double-checked and excluded.

### 2.4. Data Collection

After finalizing the included studies, bibliographic details of studies focused on ophthalmic features of COVID-19 were retrieved. We updated the bibliographic details using Endnote's “find references updates” feature and then extracted the proposed details, which included the name of the first author, the country where the first author was affiliated, the study type, the year and the month of publication, the journal along with its Web of Science impact factor and Scopus cite score, and the language, using Microsoft Office Excel 2019 built-in Power Query (Microsoft Corp. Redmond, WA). Next, we manually checked for missing data and completed the data as far as we could. Data were then manually checked and confirmed by 2 reviewers (AF and VM), with discrepancies being resolved by discussion and involvement of a third reviewer when necessary. Articles with inaccessible data were included in all analyses and reported as “not mentioned/accessible.”

### 2.5. Quality Assessment

The quality of the design and reporting of the included studies was assessed using the Joanna Briggs Institute (JBI) critical appraisal checklist according to the type of studies (including cross-sections, case-controls, case reports, case series, qualitative research, randomized controlled trials, systematic reviews) [5].

### 2.6. Data Analysis

The categorical data were reported as frequencies and percentages. Given that the number of publications by each country could be influenced by the population size and COVID-19 infection and mortality rate in that country, we adjusted the outputs depending on each country's population size, COVID-19 infection rates, and the mortality rate attributed to COVID-19. Adjusted data for population size were calculated as the number of publications divided by the corresponding country's population. Adjusted data for COVID-19 infection rate were calculated as the number of publications divided by the corresponding country's confirmed COVID-19 cases. Adjusted data for COVID-19 mortality were calculated as the number of publications divided by the corresponding country's COVID-19 deaths. Data on country populations were obtained from the World Bank [6]. Data on the total number of confirmed COVID-19 cases and deaths in each country were retrieved from Our World in Data on 2^nd^ April, 2021 [7].

All data used in this study were publicly available; thus, Institutional Review Board (IRB)/Ethics Committee approval was not required. The study adhered to the tenets of the Declaration of Helsinki.

## 3. Results

**Figure 1:**
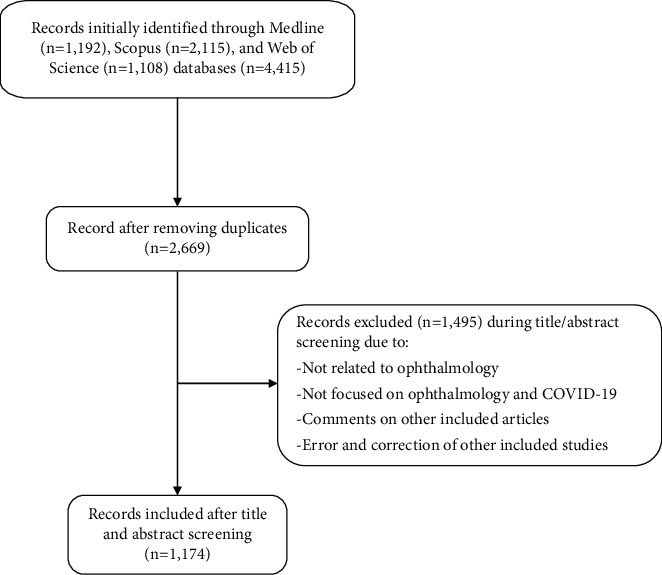
Flowchart of the article selection process (COVID-19: Coronavirus disease).

Following systematic searching of PubMed (1,192), Scopus (2,115), and Web of Science (1,108), a total of 4,415 articles were identified. After removing the duplicates, 2,669 distinct articles were screened based on their title and abstract. Of these, 1,495 articles were excluded. Finally, 1,174 ophthalmology-focused articles were included in the analysis. The flowchart of article selection is shown in Figure 1. The comparison of the number of all included articles with the number of all COVID-19-related articles (approximately about 238,618 at the time of the study [8]) showed that ophthalmology-focused publications (1,174 articles) accounted for less than 0.5 percent of the total articles.

Most of the articles were published in the Indian Journal of Ophthalmology (121 articles), Eye (43 articles), European Journal of Ophthalmology (37 articles), and Graefe's Archive for Clinical and Experimental Ophthalmology (37 articles) (Table 1).

The most frequent (507 out of 1,174) study design was original article. Other common types were review article (*n* = 214, 18%) and letter to the editor (*n* = 189, 16%) (Table 2). Regarding the linguistic aspect, most of the articles were in English (88.25%; Table 3).

**Figure 2:**
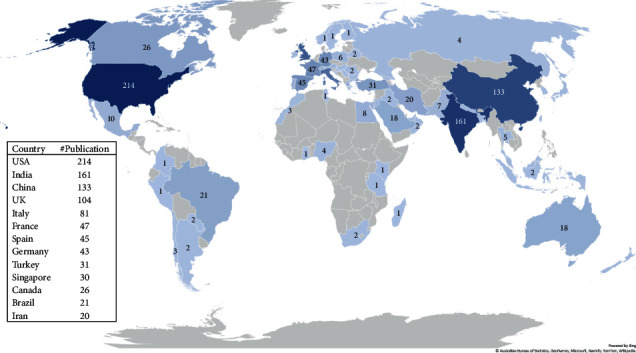
The worldwide distribution of publications across the countries. Countries with higher than 20 publications were shown in the box (USA: United States of America, UK: United Kingdom).

We analyzed the articles according to their first authors along with their affiliated countries. The United States of America (*n* = 214), India (*n* = 161), China (*n* = 133), and the United Kingdom (*n* = 104) were the countries with the highest number of publications (Figure 2). The article counts were also adjusted according to the country's population size, COVID-19 infection rates, and mortality attributed to COVID-19. Singapore and the United Kingdom were the countries with the highest number of publications based on their total population. Due to the early control of COVID-19 infection and the rapid attenuation in the mortality rate in China, the adjusted publication counts for COVID-19 confirmed cases and deaths in this country were much higher than those in other countries (Supplementary Material 2)

The percentage of publications whose first authors had equal to or higher than 3 publications were among the highest (therefore more centralized) in Germany and France (Supplementary Material 3)

**Figure 3:**
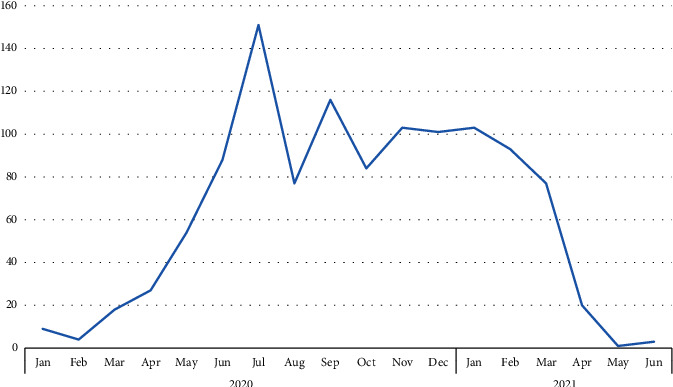
The number of publications by the year and month they were published.

The number of publications in each month during the years 2020 and 2021 is presented in Figure 3. It seems that the publication rate during the initial months of 2021 faced a decline compared with the middle and last months of 2020. The accumulative frequency of the publications in 2020 and 2021 were 872 and 302 articles, respectively.

After analyzing the bibliometric aspects of ophthalmology-focused scientific literature on the COVID-19 disease, we highlighted the most common issues in the field, which are discussed in separate sections.

### 3.1. Ocular Tropism, Receptors, and Diagnosis

Similar to other members of the coronavirus family, the main type of SARS-CoV-2 transmission is through infected droplets during close contact [9]; nevertheless, several other possible transmission routes have also been described [10, 11]. Eyes have been mentioned as potential sites for the virus entry. The SARS-CoV-2 virus may be transmitted to the ocular surface via hands, contact lenses, and droplets. Different hypotheses were formed to suggest the possibility of ophthalmic transmission through the following mechanisms: (1) direct entry of the virus to the conjunctiva; (2) transportation of the virus from the ocular surface to the upper respiratory tract; and (3) hematogenic infection via the tear gland [9].

Different molecular domains exist on the ocular surface for SARS-CoV-2 entry, including ACE2, TMPRSS2, and CD147 cathepsin L (CTSL) [12], while several protective elements were also described (e.g., existence of lipocalin, lactoferrin, immunoglobulin A (IgA), and lysozyme) [13]. The required amount of virus particles and receptor expression needed to cause infection has not been determined yet [14]. Although the natural expression of SARS-CoV-2 receptors in the eye is too low in comparison with other tissues [13], SARS-CoV-2 may utilize proinflammatory signals (TNF, NFK*β*, and IFN-*γ*) to upregulate the expression of ACE2 and TMPRSS2 in the superficial conjunctival epithelium for an enhanced entry [15]. On the other hand, the underlying ocular surface disease could make the patient prone to virus entry through the disrupted defensive mechanisms of the eye surface [16].

The rate of positive RT-PCR (real-time polymerase chain reaction) tests for conjunctival swabs in confirmed COVID-19 patients has been reported from 0% to 57.1% in different studies [9, 17–21]. This wide range can be justified by the varying levels of disease severity, the variety in sampling time and the methods used, multiple sample sizes, and also different sensitivities of the RT-PCR kits [22]. Although the rate of virus detection by conjunctival swabs RT-PCR in confirmed patients was significantly lower compared with the other diagnostic modalities [9, 17–21], which was probably due to the dilution and washing out of viruses via the tear fluid [22], the infectivity of the detected viruses was still confirmed [17]. Notably, it has been shown that the existence of conjunctivitis is not a necessary factor for SAR-CoV-2 detection in these samples [17]. The existence of the SARS-CoV-2 virus in inner ocular tissues was also confirmed in alternate studies [23, 24]. These aforementioned pieces of evidence support the probability of SARS-CoV-2 ocular transmission [25], but controversial issues still remain [13, 17].

In a meta-analysis by Ulhaq and Soraya, the specificity of ocular fluid and tissues was 100%, but the sensitivity for detecting SAR-CoV-2 was extremely low, at about 0.6% [26]. Due to this much lower sensitivity in comparison with nasal samples, the use of the tear and the conjunctival swab is not a reliable diagnostic method. Negative nasopharyngeal and positive conjunctival swab test results in some COVID-19 patients suggested that slightly invasive conjunctival sampling could be considered a complementary diagnostic method [21, 27].

### 3.2. Ophthalmic Manifestations

An uncontrolled case series of Brazilian newborns of mothers with COVID-19 infection showed that none of the babies with a COVID-19-positive PCR test had ocular manifestations [28]. However, while this particular report may refute the association between SARS-CoV-2 and ophthalmic manifestations, many other studies have reported this association.

According to the study by Deiner et al., online search interest in ocular symptoms (representing conjunctivitis) increased during the pandemic compared with that the same period of the previous year (especially in Italian society), suggesting the possible effect of COVID-19 infection on the eyes [29].

Studies showed that the presentation, timing, and severity of COVID-19 ocular manifestations differ among patients. These manifestations are usually seen in severe diseases accompanying systemic signs and abnormal blood parameters [30–32]. In several review papers, the frequency of various ophthalmic presentations was reported to be low (from 0 to 31.58%) [20, 22, 33], and in three meta-analysis studies, the calculated frequency was almost 11% [34–36]. Variability in reported rates between studies could be due to the confounding effect of unobserved factors [37]. Sometimes it is challenging to determine which ophthalmic manifestation is caused by the virus directly and which one is related to secondary immune response, proinflammatory status, or coagulopathies [38].

All parts of the eye can be relatively affected by the virus, which leads to various ophthalmic presentations. It has been reported that these presentations can develop in any stage of the disease, even as the initial symptom in COVID-19 patients (1% to 12.5%) [35, 39, 40]. Neuro-ophthalmic abnormalities usually are revealed 5 days after manifestation of systemic symptoms, but presentations related to the ocular surface and anterior segment are often observed 8.5 days after the initiation of the symptoms; posterior segment and orbital pathologies will appear 12 days after the diagnosis of COVID-19 [38].

The prevalence of eyelid, ocular surface, and anterior segment-related symptoms in different studies has been reported at a range from 0.81% to 34.5% [38]. Conjunctivitis is the most common ophthalmic manifestation and can be observed in different stages of the disease [34, 38]. It seems to be self‐limiting and can be resolved without any specific treatment [41]. Also, conjunctivitis can be the result of multisystem inflammatory syndrome in children (MIS-C), which is known as a Kawasaki-like condition [38]. Some of the other common ocular surface symptoms are dryness (6.9–37%), pain (10.3–31.2%), discharge (6.9–29.6%), redness (10.8–24.1%), and foreign body sensation (6–18.5%) [38]. A study reported that eye dryness persisted in 12% of patients even 15 days after RT-PCR SARS-CoV-2 negativity [42]. Manifestations of COVID-19 in the posterior segment of the eye include retinal vascular abnormalities like central retinal artery occlusion (CRAO), retinal vein occlusion (RVO), and choroiditis, along with retinal findings. A cross-sectional study reported retinal changes in 55.6% of patients admitted with severe COVID-19, mainly flame-shaped hemorrhages and ischemic pattern lesions (cotton wool spots and retinal sectoral pallor) [43]. Also, neuro-ophthalmic signs (like papillophlebitis, Adie's tonic pupil, and optic neuritis) and orbital manifestations (such as dacryoadenitis, retro-orbital pain, mucormycosis, orbital cellulitis, and sinusitis) have been reported [38].

### 3.3. Prevention

A cohort study of hospitalized patients of Suizhou, China, demonstrated that wearing eyeglasses for more than 8 hours a day has a protective effect against COVID-19 infection [44]. Beyond using the eyeglasses, some topical medications with antiviral properties can prevent SARS-CoV-2 attachment, entry, and replication in ocular tissues. These topical drugs include chloroquine, trehalose, antihistamines, and interferons, all of which have safety approvals in ophthalmological practice [45]. Local use of the combination of chloroquine, zinc, and azithromycin has been suggested to have a prophylactic effect even after exposure to the viral particles [46].

#### 3.3.1. Outpatient Clinic

Infection by COVID-19 is a risk for all physicians; however, some factors make ophthalmologists more susceptible. Proximity to patients and direct contact with conjunctival mucosal surfaces can make them more prone to COVID-19 infection.

Studies have shown that droplets can spread over 2 meters at a speed of up to 50 m/s during coughing or sneezing [47]; therefore, they can contaminate environmental surfaces, especially the medical devices shared for ophthalmology examination [48]. Before ophthalmologic visits, all patients should be assessed for SARS-CoV-2 infection during a reliable triage process, which can prevent unintentional contact with the virus. Triaged patients must be kept in a waiting room with favorable air conditioning and maximum distance between the patients [49]. However, due to false-negative results, asymptomatic COVID-19 patients may pass triage and contaminate the ophthalmology examination room [50]; therefore, taking proper personal protective equipment (PPE) and decontaminating the examination room after visits are essential. Current clinical practice guidelines support the idea of continuing mask wear by ophthalmologists during all patient encounters, even after vaccination [47].

Clinicians should be aware of touching the eyelids and corneal surface with the tip of the eye drop bottles like mydriatic drops usually used during eye examinations. The physician's hands also should be disinfected immediately after the procedure [47]. During the slit-lamp examination, using facial masks by both patients and physicians is crucial in decreasing aerosols. Using both a face mask and slit-lamp shield leads to the least dispersion of the virus [51]. Slit-lamp shields have a barrier role against large droplets, but their protection against smaller ones is not proven. Large slit-lamp shields should be placed close to the patient and routinely disinfected [52]. As this approach on its own cannot prevent contamination of equipment and surfaces, they should also be decontaminated following every visit [47]. Due to the increased risk of virus spread, any conversation must be avoided as much as possible during the slit-lamp examinations [47].

Using disposable single-use tools like tonometer tips is another means to reduce the spread of viral particles [47]. Noncontact tonometer devices are not as safe as we thought they would be and must be avoided due to the creation of microaerosols that can disperse the virus [53]. Disinfectant solutions containing 70% alcohol could be used for the disinfection of surfaces and devices (e.g., tonometer tips), while other diagnostic equipment such as visual field analyzers should be disinfected according to the manufacturer's instructions [47].

Ocular irritation should be avoided by physicians as much as possible. It can stimulate patients to rub their eyes with contaminated hands. If it is not avoidable, the patient must be warned about the increased risk of infection through hand-to-face contact [54].

Ophthalmologists should postpone the assessment of confirmed or highly suspected patients, and in case of the need for urgent assessment, they should use optimum PPE, including respiratory masks (FFP2/N95), waterproof long-sleeved gowns, face shields, and gloves, to reduce the risk [49]. During outpatient procedures that require proximity to patients, like intravitreal injection or lateral tarsorrhaphy, the patient should wear a surgical mask or a cloth face covering. Also, ophthalmologists are recommended to use a surgical mask or an N95 mask if a patient is unable to wear a mask or during visits with young children, along with eye protection [47].

#### 3.3.2. Surgical Procedures

For ophthalmic surgical procedures that may generate aerosol particles, a preoperative PCR test for asymptomatic patients should be considered [47]. In addition to the routine measures, including “general infection control” and PPE usage, utilizing the least number of staff in the operation room should be considered [49]. Appropriate PPE should be chosen based on the type of surgery, patient condition, and the prevalence of COVID-19 [47]. Whenever possible, surgery should be performed under local anesthesia to avoid the generation of aerosols during general anesthesia procedures; otherwise, exposure should be minimized during intubation and extubation [49]. For minimizing the spread of respiratory droplets in procedures performed under monitored sedation, a patient's mask with a tight seal of the surgical drape could be used [47, 55]. For suspected or confirmed patients full PPE, airborne isolation in the room with negative pressure, assigning an experienced surgeon, and using the most familiar methods to shorten the time of surgery as much as possible must be considered [49]. Periocular usage of 5%–10% povidone-iodine for about 3 minutes can cause an effective reduction in the viral load of the ocular surface [56].

Vaccinated persons recently exposed to the virus do not usually develop disease symptoms, but they may be carriers and can potentially infect others. Therefore, RT-PCR testing and PPE usage should be done before surgery [47]. In patients with COVID-19 viremia, SARS-CoV-2 RNA was detected in the corneal tissue [57]. As a result, some articles have suggested excluding the SARS-CoV-2-infected donor tissues for transplantation [58, 59]. However, the Eye Bank Association of America (EBAA) and Food and Drug Association (FDA) have not recommended asymptomatic PCR screening. Up to now, there has not been any reported case of COVID-19 transmission through transplantation [60, 61].

### 3.4. Hand Sanitizer and Ocular Injury

Multiple studies reported the increasing rate of accidental ocular injuries in the pediatric population due to contact with alcohol-based hand sanitizers (ABHSs). A retrospective study by Martin et al. in France demonstrated that the number of ABHS-related ocular injuries in children during the pandemic had increased 7 times compared with the previous year. Thirteen percent of the mentioned injuries were severe and required surgical intervention. Several preventative measures could be implemented to prevent such events: replacement of ABHSs with soap and water especially at home, having suitable dispensers for children (at a lower height and below the face level) with cautionary signs, and training children in using ABHSs [62, 63].

### 3.5. Myopia Progression

Based on a prospective cross-sectional study by Wang et al., it was shown that home confinement during the pandemic era is associated with a significant myopic shift, especially in 6 to 8-year-old children (approximately −0.3 diopters). The prevalence of myopia in the 2020 screenings was significantly higher than the highest prevalence of myopia within 2015–2019 for 6 to 8-year-old children, indicating the lockdown effect of COVID-19 on vision [64]. The leading causes of myopia progression have been attributed to increased screen time and decreased outdoor activity [65].

### 3.6. Ocular Complications and COVID-19 Treatment Strategies

During the SARS-CoV-2 pandemic, several drugs were considered effective in treating or preventing the progression of the disease. Chloroquine/hydroxychloroquine, systemic corticosteroids, intravenous immunoglobulin (IVIG), and antiviral agents were the most common. Some of which have proven ophthalmic side effects.

During the first days of the pandemic, chloroquine or hydroxychloroquine were among the few treatments for COVID-19. Although long-term use of chloroquine or hydroxychloroquine was known to cause retinal toxicity, it was not observed within the short period of use for COVID-19 [66]. Therefore, routine baseline ophthalmic examination would not be necessary if appropriate dosages were chosen. Ophthalmic evaluation should be considered for patients with a previous history or pre-existing maculopathy due to ophthalmic concerns following the use of these aforementioned drugs [67–69].

Systemic corticosteroids are widely used in COVID-19 management as a protective agent against the severe immune response, but these drugs can lead to cataract, glaucoma, and central serous chorioretinopathy. Another noticeable adverse particularly found in predisposed patients is the increased risk of life-threatening opportunistic infections like fungal organisms (rhino-orbito-cerebral mucormycosis), which needs a prompt development of a guideline for prophylactic use of antifungals in patients with risk factors [38]. Receiving IVIG to modulate immune response may lead to central retinal vein occlusion (CRVO), which is rarely reported in patients [38].

Lopinavir and ritonavir, protease inhibitor agents which have controversial treatment effects on COVID-19, may cause reactivation of autoimmune conditions. Due to the retinal toxicity of ritonavir, it has been hypothesized that simultaneous use of it with chloroquine/hydroxychloroquine can lead to a synergic toxic effect on the retina [70, 71]. Ribavirin, another antiviral agent, has not been used much for COVID-19 but can cause major ophthalmologic complications such as retinopathy, retinal vein occlusion, and serous retinal detachment [38].

Interferons (e.g., alfa, beta) as other favorable medications have been associated with retinopathy, Vogt–Koyanagi–Harada disease, optic neuropathy, etc. Tocilizumab (anti-IL-6) as an immunosuppressive agent with positive effects on COVID-19 treatment could cause cotton wool spots and hemorrhages in the retina [38].

Prone positioning is a way to improve oxygenation in critically ill COVID-19 patients who are unresponsive to optimal ventilator settings. A prolonged period of prone positioning can lead to orbital compartment syndrome, which can be avoided by cushioning around the eyes while maintaining the patient's head position above the heart level [72].

### 3.7. COVID-19 Effect on Ophthalmologic Care and Operations

Like other disciplines, interruption in follow-ups is considered a serious problem in ophthalmology, especially in chronic diseases. The rate of interruptions has increased 4 times more than before due to COVID-19-related concerns among the patients [73]. Novel treatment strategies should be introduced to make follow-ups more feasible during pandemics. On the other hand, despite the American Association of Ophthalmology's recommendation to continue performing urgent or emergent vitreoretinal surgical procedures, the frequency of such procedures declined and remained at a low level even after the COVID-19 pandemic peaks. The main outcomes of this decrease have not been fully understood [74]. A study conducted in Italy reported that urgent surgeries and intravitreal injections were reduced by almost 50% in the lockdown period compared with control periods [75]. Delay in intravitreal injections of anti-vascular endothelial growth factors (anti-VEGFs) as the main treatment for retinal vascular abnormalities can lead to vision impairment. Billioti de Gage et al. reported a relative decline in anti-VEGF injections in France [76]; the actual burden of which should be evaluated in the future. For the best management, anti-VEGF treatment should be simplified, and patients at higher risk of permanent visual loss should be prioritized [77, 78].

### 3.8. Telemedicine

The importance of telemedicine has been comprehended during the COVID-19 era more than before. Ophthalmology was a pioneer in employing technology for this purpose. Virtual clinics and online video consulting showed that novel telemedicine tools could be used as effective strategies for delivering ophthalmic care services with a high acceptance rate (about 86.1%) among the patients [79, 80]. Moreover, artificial intelligence (AI) has been used to detect and screen retinal pathologies like diabetic retinopathy, macular edema, AMD, and retinopathy of prematurity [81, 82]. Also, digital self-monitoring devices (such as iCare HOME and implantable IOP-sensors for intraocular pressure measurements, ForeseeHome™ for measurement of preferential hyperacuity perimetry) and smartphone applications (Alleye™ for hyperacuity testing, MyVisionTrack™ for shape discrimination hyperacuity) can be used at home for ophthalmic diseases such as AMD [83–85]. Mechanized slit-lamps with live audio/video and stereo-viewing capabilities and the ability to acquire diagnostic images with smartphone cameras are considered novel strategies in examining the anterior segment of the eye [86].

Some limitations have been described regarding using these novel approaches in telemedicine, including unfamiliarity with the structure of these devices and applications for both the patient and physician, concerns regarding their accuracy and reliability, cost, technical problems like Internet interruption, and also policy and privacy issues. These issues should be resolved before their successful extensive application [87].

### 3.9. Ophthalmology Training

During the pandemic, the number of outpatient visits significantly reduced, and elective surgeries were suspended to prevent the spreading of the virus. This has led to a notable reduction in educational activities related to medical sciences disciplines, including ophthalmology [88]. Disease breakout has had a notable impact on surgical training for residents, and traveling restrictions make alternative surgical sites impossible to access [89]. A survey that was held in India demonstrated that nearly 80.7% of the ophthalmology trainees felt that the COVID-19 crisis had a negative impact on their surgical training [90]. To reduce the negative impact of the disease on training, some alternative ways like video databases, simulators, and dry/wet lab models are designed to help residents learn surgical skills [88].

A study reported that the most challenging issue of both forms of distance education, i.e., online and offline teaching, is the lack of interaction between teachers and students and also among students themselves. The combination of online live classes and offline prerecorded videos are recommended, but the low level of interaction can still seriously attenuate the training quality and should be resolved as soon as possible [91].

## 4. Discussion

As the pandemic spreads, an increasing number of research articles addressing various medical topics are being published. Scientific and medical research is critical for understanding different aspects of COVID-19 and its association with different human body organs. Finding current needs and future directions in ocular research during this pandemic is essential.

We reviewed the current literature regarding the major topics, including SARS-CoV-2 ocular tropism, ophthalmic manifestations, prevention, effects of lockdown and sanitizers on ocular health, ocular complications of COVID-19 treatment strategies, eye care services during the pandemic, telemedicine, and ophthalmology training.

Our bibliometric analysis revealed that ophthalmic-related publications accounted for less than 0.5 percent of the total articles. Most of the articles were original articles in English. It seems that the publication rate during the initial months of 2021 faced a decline compared with the middle and last months of 2020. The USA is the most affiliated country; on the other hand, Singapore and the United Kingdom were the countries with the highest number of population-adjusted publications.

The number of bibliometric analyses of ophthalmologic-focused COVID-19 articles is very limited. We found only two similar studies, in which only the PubMed database with a much shorter period was searched [92, 93]. They also did not provide a review of the major subjects.

This review has some strengths and limitations. According to the high production rate of COVID-19-related studies, conducting such studies requires multiple search updates throughout the time, and as the main limitation, we may not have included the recently published articles in this review. In the present study, the bibliometric aspect of all ophthalmology-focused COVID-19 publications was analyzed, and a comprehensive review of major topics was done. Systematic searching and screening of publications using three large databases (PubMed, Scopus, and Web of Science) contributed to achieving more reliable results.

In conclusion, our study highlighted the small number of ophthalmology-related publications in the COVID-19 research field. Also, the comprehensive review of the major research topics in this field revealed some controversies. Due to the limited proportion of these studies and the mentioned controversies, we recommend further global scientific studies to create a brighter sketch of this field. As the pandemic continues, considering what we have learned throughout this time could help us direct future ophthalmology changes, especially amid similar conditions.

## Figures and Tables

**Table 1:** The list of top 30 journals that published ophthalmology-focused articles, their Web of Science impact factor, CiteScore, and the number of publications.

Journal (NLM abbreviated)	Impact factor	CiteScore	Number of publications
Indian J Ophthalmol	1.250	1.6	121
Eye (Lond)	2.455	23.4	43
Eur J Ophthalmol	1.642	2.7	37
Graefes Arch Clin Exp Ophthalmol	2.396	4.2	37
JAMA Ophthalmol	6.198	9	30
Ophthalmology	8.470	14.8	29
J Fr Ophtalmol	0.636	0.6	28
Ocul Immunol Inflamm	2.112	3.1	25
Clin Ophthalmol	2.832	4.4	24
Ophthalmologe	0.698	1.1	22
Am J Ophthalmol	4.013	7.7	22
Curr Opin Ophthalmol	2.983	5.3	16
Chinese Journal of Experimental Ophthalmology	0.020	0.4	15
J Med Virol	2.021	4	15
Cont Lens Anterior Eye	2.578	3.4	14
Can J Ophthalmol	1.369	1.6	14
J Glaucoma	1.992	3.5	14
Acta Ophthalmol	3.362	4.8	14
J Cataract Refract Surg	2.689	4.2	14
Br J Ophthalmol	3.611	6.8	13
International Eye Science	0.040	0.1	12
Cornea	2.215	4	11
Arch Soc Esp Oftalmol	0.420	0.7	11
Ophthalmol Ther	3.000	2.9	10
Asia Pac J Ophthalmol (Phila)	2.250	4.2	10
Int Ophthalmol	1.33	1.8	10
Cureus	N/A	N/A	9
Community Eye Health	N/A	0.1	9
Ophthalmic Plast Reconstr Surg	1.331	1.7	9
Am J Ophthalmol Case Rep	0.480	0.8	9

NLM: National Library of Medicine, N/A: not applicable.

**Table 2:** The distribution of publications by their type of publication.

Type of publication	Number of publications	Percentage
Original article	507	43
Review	214	18
Letter to editor	189	16
Editorial	117	10
Case Report	55	5
Note	41	3
Practice guideline	19	2
Report	12	1
Others^*∗*^	20	2

^*∗*^Others: correspondence (*n* = 5), communications (*n* = 5), short survey (*n* = 3), abstract (*n* = 2), conference paper (*n* = 1), book chapter (*n* = 1), and not mentioned/accessible (*n* = 3).

**Table 3:** The distribution of ophthalmology-focused articles by their publication language.

Languages	Number of publications	Percentage
English	1036	88.25
Chinese	41	3.49
English; French	23	1.96
German	22	1.87
English; Spanish	19	1.62
Spanish	10	0.85
French	8	0.68
Others^*∗*^	15	1.28

^*∗*^Others: Russian (*n* = 6), Portuguese (*n* = 2), English; German (*n* = 1), English; Turkish (*n* = 1), Hungarian (*n* = 1), Japanese (*n* = 1), Korean (*n* = 1), and not mentioned/accessible (*n* = 2).

## Data Availability

The data that support the findings of this study are available on request from the corresponding author [Ali Forouhari].

## Supplementary Materials

Supplementary Materials Supplementary Material 1. Search strategies Supplementary Material 2. Top ten countries regarding the number of publications per 100,000 population, per 100,000 cases of COVID-19, and per 100,000 deaths due to COVID-19. Supplementary Material 3. First authors with three or more publications by their affiliated countries.
