# Supplementary material for: A Systematic Literature Review and Bibliometric Analysis of Ophthalmology and COVID-19 Research
Source: J Ophthalmol. 2022 May 24;2022:8195228. doi: 10.1155/2022/8195228 (PMC9133895; doi:10.1155/2022/8195228)
Supplement: Supplementary Materials — Supplementary Material 1. Search strategies Supplementary Material 2. Top ten countries regarding the number of publications per 100,000 population, per 100,000 cases of COVID-19, and per 100,000 deaths due to COVID-19. Supplementary Material 3. First authors with three or more publications by their affiliated countries. [file 8195228.f1.zip › 8195228.f1/Supp 1.docx]

Supplementary Material 1: Search strategies

**PubMed**

("Eye"[MeSH Terms] OR "ophthalmology"[MeSH Terms] OR "Eye Diseases"[MeSH Terms] OR "ocular manifestation*"[Title/Abstract] OR "ocular symptom*"[Title/Abstract] OR "ophthalmic manifestation*"[Title/Abstract] OR "eye manifestation*"[Title/Abstract] OR "ophthalmologist*"[Title/Abstract] OR "ocular*"[Title/Abstract] OR "conjunctivitis*"[Title/Abstract] OR "conjunctiva*"[Title/Abstract] OR "slit lamp*"[Title/Abstract] OR "slit lamp*"[Title/Abstract] OR "ophthalmic*"[Title/Abstract] OR "eye infection*"[Title/Abstract] OR "ocular infection*"[Title/Abstract] OR "eye disorder*"[Title/Abstract]) AND ("covid 19"[Title/Abstract] OR "covid 19 virus disease"[Title/Abstract] OR "covid 19 virus disease"[Title/Abstract] OR "covid 19"[Title/Abstract] OR ("Disease"[Title/Abstract] AND "covid 19 virus"[Title/Abstract]) OR ("Virus Disease"[Title/Abstract] AND "covid 19"[Title/Abstract]) OR "covid 19 virus infection"[Title/Abstract] OR "covid 19 virus infection"[Title/Abstract] OR "COVID-19 Virus Infections"[Title/Abstract] OR ("Infection"[Title/Abstract] AND "covid 19 virus"[Title/Abstract]) OR ("Virus Infection"[Title/Abstract] AND "covid 19"[Title/Abstract]) OR "2019 ncov infection"[Title/Abstract] OR "2019 ncov infection"[Title/Abstract] OR "2019-nCoV Infections"[Title/Abstract] OR ("Infection"[Title/Abstract] AND "2019-nCoV"[Title/Abstract]) OR "coronavirus disease 19"[Title/Abstract] OR "coronavirus disease 19"[Title/Abstract] OR "2019 Novel Coronavirus Disease"[Title/Abstract] OR "2019 Novel Coronavirus Infection"[Title/Abstract] OR "2019 ncov disease"[Title/Abstract] OR "2019 ncov disease"[Title/Abstract] OR "2019-nCoV Diseases"[Title/Abstract] OR ("Disease"[Title/Abstract] AND "2019-nCoV"[Title/Abstract]) OR "COVID19"[Title/Abstract] OR "Coronavirus Disease 2019"[Title/Abstract] OR ("Disease 2019"[Title/Abstract] AND "Coronavirus"[Title/Abstract]) OR "SARS Coronavirus 2 Infection"[Title/Abstract] OR "sars cov 2 infection"[Title/Abstract] OR ("Infection"[Title/Abstract] AND "SARS-CoV-2"[Title/Abstract]) OR "sars cov 2 infection"[Title/Abstract] OR "SARS-CoV-2 Infections"[Title/Abstract] OR "covid 19 pandemic"[Title/Abstract] OR "covid 19 pandemic"[Title/Abstract] OR "COVID-19 Pandemics"[Title/Abstract] OR ("Pandemic"[Title/Abstract] AND "covid 19"[Title/Abstract]) OR "Coronavirus Disease 2019 Virus"[Title/Abstract] OR "2019 Novel Coronavirus"[Title/Abstract] OR "2019 Novel Coronaviruses"[Title/Abstract] OR ("Coronavirus"[Title/Abstract] AND "2019 Novel"[Title/Abstract]) OR "Wuhan Seafood Market Pneumonia Virus"[Title/Abstract] OR "sars cov 2 virus"[Title/Abstract] OR "sars cov 2 virus"[Title/Abstract] OR "SARS-CoV-2 Viruses"[Title/Abstract] OR ("Virus"[Title/Abstract] AND "SARS-CoV-2"[Title/Abstract]) OR "2019-nCoV"[Title/Abstract] OR "covid 19 virus"[Title/Abstract] OR "covid 19 virus"[Title/Abstract] OR "COVID-19 Viruses"[Title/Abstract] OR ("Virus"[Title/Abstract] AND "covid 19"[Title/Abstract]) OR "Wuhan Coronavirus"[Title/Abstract] OR ("Coronavirus"[Title/Abstract] AND "Wuhan"[Title/Abstract]) OR "SARS Coronavirus 2"[Title/Abstract] OR ("Coronavirus 2"[Title/Abstract] AND "SARS"[Title/Abstract]) OR "Severe Acute Respiratory Syndrome Coronavirus 2"[Title/Abstract] OR "SARS-CoV-2"[MeSH Terms] OR "covid 19"[MeSH Terms])

**Scopus**

(TITLE-ABS-KEY("COVID 19") OR TITLE-ABS-KEY("COVID-19") OR TITLE-ABS-KEY("COVID-19 Virus Disease") OR TITLE-ABS-KEY("COVID 19 Virus Disease") OR TITLE-ABS-KEY("COVID-19 Virus Diseases") OR (TITLE-ABS-KEY(Disease) AND TITLE-ABS-KEY("COVID-19 Virus")) OR (TITLE-ABS-KEY("Virus Disease") AND TITLE-ABS-KEY("COVID-19")) OR TITLE-ABS-KEY("COVID-19 Virus Infection") OR TITLE-ABS-KEY("COVID 19 Virus Infection") OR TITLE-ABS-KEY("COVID-19 Virus Infections") OR (TITLE-ABS-KEY(Infection) AND TITLE-ABS-KEY("COVID-19 Virus")) OR (TITLE-ABS-KEY("Virus Infection") AND TITLE-ABS-KEY("COVID-19")) OR TITLE-ABS-KEY("2019-nCoV Infection") OR TITLE-ABS-KEY("2019 nCoV Infection") OR TITLE-ABS-KEY("2019-nCoV Infections") OR (TITLE-ABS-KEY(Infection) AND TITLE-ABS-KEY("2019-nCoV")) OR TITLE-ABS-KEY("Coronavirus Disease-19") OR TITLE-ABS-KEY("Coronavirus Disease 19") OR TITLE-ABS-KEY("2019 Novel Coronavirus Disease") OR TITLE-ABS-KEY("2019 Novel Coronavirus Infection") OR TITLE-ABS-KEY("2019-nCoV Disease") OR TITLE-ABS-KEY("2019 nCoV Disease") OR TITLE-ABS-KEY("2019-nCoV Diseases") OR (TITLE-ABS-KEY(Disease) AND TITLE-ABS-KEY("2019-nCoV")) OR TITLE-ABS-KEY("COVID19") OR TITLE-ABS-KEY("Coronavirus Disease 2019") OR (TITLE-ABS-KEY("Disease 2019") AND TITLE-ABS-KEY(Coronavirus)) OR TITLE-ABS-KEY("SARS Coronavirus 2 Infection") OR TITLE-ABS-KEY("SARS-CoV-2 Infection") OR (TITLE-ABS-KEY(Infection) AND TITLE-ABS-KEY("SARS-CoV-2")) OR TITLE-ABS-KEY("SARS CoV 2 Infection") OR TITLE-ABS-KEY("SARS-CoV-2 Infections") OR TITLE-ABS-KEY("COVID-19 Pandemic") OR TITLE-ABS-KEY("COVID 19 Pandemic") OR TITLE-ABS-KEY("COVID-19 Pandemics") OR (TITLE-ABS-KEY(Pandemic) AND TITLE-ABS-KEY("COVID-19")) OR TITLE-ABS-KEY("Coronavirus Disease 2019 Virus") OR TITLE-ABS-KEY("2019 Novel Coronavirus") OR TITLE-ABS-KEY("2019 Novel Coronaviruses") OR (TITLE-ABS-KEY(Coronavirus) AND TITLE-ABS-KEY("2019 Novel")) OR TITLE-ABS-KEY("Wuhan Seafood Market Pneumonia Virus") OR TITLE-ABS-KEY("SARS-CoV-2 Virus") OR TITLE-ABS-KEY("SARS CoV 2 Virus") OR TITLE-ABS-KEY("SARS-CoV-2 Viruses") OR (TITLE-ABS-KEY(Virus) AND TITLE-ABS-KEY("SARS-CoV-2")) OR TITLE-ABS-KEY("2019-nCoV") OR TITLE-ABS-KEY("COVID-19 Virus") OR TITLE-ABS-KEY("COVID 19 Virus") OR TITLE-ABS-KEY("COVID-19 Viruses") OR (TITLE-ABS-KEY(Virus) AND TITLE-ABS-KEY("COVID-19")) OR TITLE-ABS-KEY("Wuhan Coronavirus") OR (TITLE-ABS-KEY(Coronavirus) AND TITLE-ABS-KEY(Wuhan)) OR TITLE-ABS-KEY("SARS Coronavirus 2") OR (TITLE-ABS-KEY("Coronavirus 2") AND TITLE-ABS-KEY(SARS)) OR TITLE-ABS-KEY("Severe Acute Respiratory Syndrome Coronavirus 2")) AND (TITLE-ABS-KEY("ophthalmologist*") OR TITLE-ABS-KEY("ocular*") OR TITLE-ABS-KEY("conjunctivitis*") OR TITLE-ABS-KEY("conjunctiva*") OR TITLE-ABS-KEY("slit-lamp*") OR TITLE-ABS-KEY("Slit lamp*") OR TITLE-ABS-KEY("Ophthalmic*") OR TITLE-ABS-KEY("eye infection*") OR TITLE-ABS-KEY("ocular infection*") OR TITLE-ABS-KEY("Eye disorder*") OR TITLE-ABS-KEY("Eye*") OR TITLE-ABS-KEY("ophthalmology*") OR TITLE-ABS-KEY("Eye Disease*")) OR TITLE-ABS-KEY("ocular manifestation*") OR TITLE-ABS-KEY("ocular symptom*") OR TITLE-ABS-KEY("ophthalmic manifestation*") OR TITLE-ABS-KEY("eye manifestation*")

**Web of Science**

(TS=(“COVID 19”) OR TS=(“COVID-19”) OR TS=(“COVID-19 Virus Disease”) OR TS=(“COVID 19 Virus Disease”) OR TS=(“COVID-19 Virus Diseases”) OR (TS=(Disease) AND TS=(“COVID-19 Virus”)) OR (TS=(“Virus Disease”) AND TS=(“COVID-19”)) OR TS=(“COVID-19 Virus Infection”) OR TS=(“COVID 19 Virus Infection”) OR TS=(“COVID-19 Virus Infections”) OR (TS=(Infection) AND TS=(“COVID-19 Virus”)) OR (TS=(“Virus Infection”) AND TS=(“COVID-19”)) OR TS=(“2019-nCoV Infection”) OR TS=(“2019 nCoV Infection”) OR TS=(“2019-nCoV Infections”) OR (TS=(Infection) AND TS=(“2019-nCoV”)) OR TS=(“Coronavirus Disease-19”) OR TS=(“Coronavirus Disease 19”) OR TS=(“2019 Novel Coronavirus Disease”) OR TS=(“2019 Novel Coronavirus Infection”) OR TS=(“2019-nCoV Disease”) OR TS=(“2019 nCoV Disease”) OR TS=(“2019-nCoV Diseases”) OR (TS=(Disease) AND TS=(“2019-nCoV”)) OR TS=(“COVID19”) OR TS=(“Coronavirus Disease 2019”) OR (TS=(“Disease 2019”) AND TS=(Coronavirus)) OR TS=(“SARS Coronavirus 2 Infection”) OR TS=(“SARS-CoV-2 Infection”) OR (TS=(Infection) AND TS=(“SARS-CoV-2”)) OR TS=(“SARS CoV 2 Infection”) OR TS=(“SARS-CoV-2 Infections”) OR TS=(“COVID-19 Pandemic”) OR TS=(“COVID 19 Pandemic”) OR TS=(“COVID-19 Pandemics”) OR (TS=(Pandemic) AND TS=(“COVID-19”)) OR TS=(“Coronavirus Disease 2019 Virus”) OR TS=(“2019 Novel Coronavirus”) OR TS=(“2019 Novel Coronaviruses”) OR (TS=(Coronavirus) AND TS=(“2019 Novel”)) OR TS=(“Wuhan Seafood Market Pneumonia Virus”) OR TS=(“SARS-CoV-2 Virus”) OR TS=(“SARS CoV 2 Virus”) OR TS=(“SARS-CoV-2 Viruses”) OR (TS=(Virus) AND TS=(“SARS-CoV-2”)) OR TS=(“2019-nCoV”) OR TS=(“COVID-19 Virus”) OR TS=(“COVID 19 Virus”) OR TS=(“COVID-19 Viruses”) OR (TS=(Virus) AND TS=(“COVID-19”)) OR TS=(“Wuhan Coronavirus”) OR (TS=(Coronavirus) AND TS=(Wuhan)) OR TS=(“SARS Coronavirus 2”) OR (TS=(“Coronavirus 2”) AND TS=(SARS)) OR TS=(“Severe Acute Respiratory Syndrome Coronavirus 2”)) AND (TS=(“eye”) OR TS=(“ophthalmology”) OR TS=(“Eye Disease”) OR TS=(“ophthalmologist”) OR TS=(“ocular”) OR TS=(“conjunctivitis”) OR TS=(“conjunctiva”) OR TS=(“slit-lamp”) OR TS=(“Slit lamp”) OR TS=(“Ophthalmic”) OR TS=(“eye infection”) OR TS=(“ocular infection”) OR TS=(“Eye disorder”) OR TS=(“eyes”) OR TS=(“Eye Diseases”) OR TS=(“ophthalmologists”) OR TS=(“eye infections”) OR TS=(“ocular infections”) OR TS=(“Eye disorders”))
