# Supplementary material for: A Systematic Literature Review and Bibliometric Analysis of Ophthalmology and COVID-19 Research
Source: J Ophthalmol. 2022 May 24;2022:8195228. doi: 10.1155/2022/8195228 (PMC9133895; doi:10.1155/2022/8195228)
Supplement: Supplementary Materials — Supplementary Material 1. Search strategies Supplementary Material 2. Top ten countries regarding the number of publications per 100,000 population, per 100,000 cases of COVID-19, and per 100,000 deaths due to COVID-19. Supplementary Material 3. First authors with three or more publications by their affiliated countries. [file 8195228.f1.zip › 8195228.f1/Supp 2.docx]

Supplementary Material 2: Top ten countries regarding the number of publications per 100,000 population, per 100,000 cases of COVID-19, and per 100,000 deaths due to COVID-19.

| **Country** | **Total population (×10^6^)** | **Number of publications per 100,000 population** | **Country** | **Total COVID-19 cases per 100,000 population** | **Number of publications per 100,000 COVID-19 cases** | **Country** | **Deaths due to COVID-19 per 100,000 population** | **Number of publications per 100,000 deaths due to COVID-19** |
| --- | --- | --- | --- | --- | --- | --- | --- | --- |
| Singapore | 5.85 | 0.512 | China | 6.98 | 15.186 | China | 0.33 | 321.212 |
| UK | 67.89 | 0.151 | India | 885.63 | 0.182 | Singapore | 0.51 | 58.824 |
| Israel | 8.66 | 0.15 | Australia | 114.92 | 0.157 | Thailand | 0.13 | 38.462 |
| Italy | 60.46 | 0.134 | Thailand | 41.35 | 0.121 | India | 11.81 | 13.633 |
| New Zealand | 4.82 | 0.104 | New Zealand | 44.48 | 0.112 | New Zealand | 0.54 | 9.259 |
| Portugal | 10.2 | 0.098 | Nigeria | 79.02 | 0.051 | Australia | 3.56 | 5.056 |
| Spain | 46.75 | 0.096 | Egypt | 197.52 | 0.041 | Nigeria | 1 | 4 |
| Switzerland | 8.65 | 0.092 | Singapore | 1032.09 | 0.029 | USA | 164.92 | 1.292 |
| France | 65.27 | 0.072 | USA | 9092.31 | 0.023 | Pakistan | 6.53 | 1.072 |
| Australia | 25.49 | 0.071 | Pakistan | 302.39 | 0.023 | Malaysia | 3.93 | 1.018 |
