# Supplementary material for: A Systematic Literature Review and Bibliometric Analysis of Ophthalmology and COVID-19 Research
Source: J Ophthalmol. 2022 May 24;2022:8195228. doi: 10.1155/2022/8195228 (PMC9133895; doi:10.1155/2022/8195228)
Supplement: Supplementary Materials — Supplementary Material 1. Search strategies Supplementary Material 2. Top ten countries regarding the number of publications per 100,000 population, per 100,000 cases of COVID-19, and per 100,000 deaths due to COVID-19. Supplementary Material 3. First authors with three or more publications by their affiliated countries. [file 8195228.f1.zip › 8195228.f1/Supp 3.docx]

Supplementary Material 3: First authors (NLM format) with three or more publications by their affiliated countries.

| **Country** | **First Author** | **Count of publications** |
| --- | --- | --- |
| **India** | **Total** | **116 (16%)** |
|  | M. J. Ali | 5 |
|  | S. K. Pandey | 4 |
|  | R. Shetty | 4 |
|  | B. Gurnani | 3 |
|  | N. Sharma | 3 |
| **France** | **Total** | **47 (27%)** |
|  | J. F. Korobelnik | 4 |
|  | H. Bourdon | 3 |
|  | V. Navel | 3 |
|  | French Society of Ophthalmology | 3 |
| **Germany** | **Total** | **43 (30%)** |
|  | T. Bayyoud | 4 |
|  | G. Deutsche Ophthalmologische | 3 |
|  | A. C. Rokohl | 3 |
|  | C. Lange | 3 |
| **Italy** | **Total** | **81 (11%)** |
|  | E. Borrelli | 3 |
|  | P. E. Napoli | 3 |
|  | M. Pellegrini | 3 |
| **Spain** | **Total** | **45 (15%)** |
|  | N. Guemes-Villahoz | 4 |
|  | M. Garcia Lorente | 3 |
| **USA** | **Total** | **214 (4%)** |
|  | T. Nanda | 3 |
|  | I. C. Kuo | 3 |
| **Saudi Arabia** | **Total** | **18 (22%)** |
|  | E. M. Almazyad | 4 |
| **Canada** | **Total** | **26 (11%)** |
|  | A. X. Nguyen | 3 |
| **UK** | **Total** | **104 (2%)** |
|  | M. Nagra | 3 |
| **Singapore** | **Total** | **30 (10%)** |
|  | D. V. Gunasekeran | 3 |
| **China** | **Total** | **133 (7%)** |
|  | S. C. L. Au | 4 |
|  | K. H. Wan | 3 |
|  | X. Li | 3 |

Values in the parentheses showed the percentages of publications whose first authors have equal to or higher than 3 publications. NLM: National Library of Medicine, UK: United Kingdom, USA: United States
